# Endurance training promotes chromatin closure and timely repression of the post-exercise immediate early stress response

**DOI:** 10.1016/j.molmet.2025.102206

**Published:** 2025-07-05

**Authors:** Laura M. de Smalen, Volkan Adak, Aurel B. Leuchtmann, Konstantin Schneider-Heieck, Stefan A. Steurer, Christoph Handschin

**Affiliations:** Biozentrum, University of Basel, Basel, Switzerland

**Keywords:** Exercise, Endurance training, Skeletal muscle plasticity, Chromatin accessibility, Immediate early genes

## Abstract

**Objectives:**

Endurance training is known to elicit numerous changes in skeletal muscle to enhance performance and function. Many of these adaptations are controlled by the modulation of transcriptional programs in myonuclei. While previous studies have explored alterations in DNA methylation and histone modifications in response to exercise, the specific changes in chromatin restructuring and accessibility, a prerequisite for transcription, are still poorly understood.

**Methods:**

A multi-omics analysis was performed: ATAC-sequencing was used to map chromatin accessibility in myonuclei isolated from endurance-trained and untrained mice at multiple time points (0 h, 6 h, and 72 h) post-exercise. Gene expression was assessed via RNA-sequencing, and motif activity analysis identified regulatory factors involved in exercise-induced chromatin remodeling and transcriptomic response.

**Results:**

Endurance training amplified rapid chromatin closing immediately after exercise, with trained muscle exhibiting a more pronounced loss of chromatin accessibility at 0 h and 6 h post-exercise compared to untrained muscle. These chromatin accessibility changes persisted longer in trained muscle, with significant retention until 72 h post-exercise. Immediate early transcription factors, such as Fos and Jun, showed a training state-dependent shift in activation dynamics. Similarly, specific modulation of genes involved in metabolism, insulin response and angiogenesis was observed.

**Conclusions:**

Endurance training triggers rapid and persistent chromatin remodeling in muscle, contributing to the transcriptional response to exercise. Our findings suggest that training induces long-lasting epigenetic changes, potentially underpinning muscle memory and improved physiological resilience. These new insights into the molecular mechanisms of muscle adaptation help to understand the training response, and might become relevant in disease prevention.

## Highlights

• Endurance training amplifies rapid chromatin closing in myonuclei following an acute bout.
• Training-induced loss of chromatin accessibility can be retained up to 72 h post-exercise.
• The immediate early stress response in trained muscle is repressed in a timely manner.

## Graphical abstract

**Figure:**
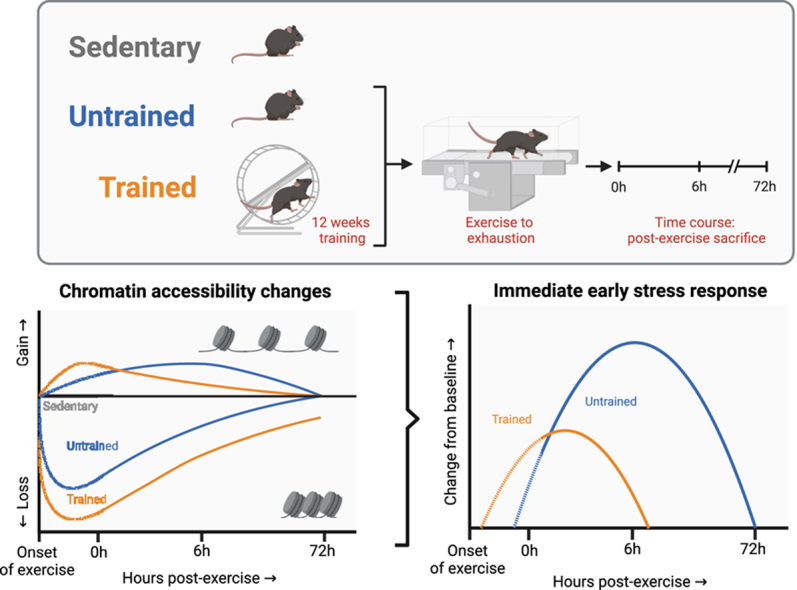

## 1 Introduction

The inherent high plasticity of skeletal muscle is demonstrated by the adaptive response to endurance exercise training, which include improved vascularization, insulin sensitivity, and mitochondrial biogenesis [1]. This array of adaptations not only facilitates and enhances the execution of future bouts of exercise, but also confers strong health benefits that extend beyond improved physical performance [1,2]. Despite these significant implications, the understanding of molecular mechanisms that drive these changes is limited. Long-term training adaptations are brought about by repeated perturbations of homeostasis evoked by individual acute exercise bouts. A large number of signals contribute to the control of exercise-induced transcriptional programs. For example, changes in energy substrate availability, calcium homeostasis and oxygen levels during exercise are sensed and integrated to activate signaling cascades, which in turn induce specific transcriptional programs and, ultimately, the expression of effector proteins that control the adaptive changes [3,4]. On the transcriptional level, exercise elicits a remarkable burst of gene activity from the onset of exercise until several hours post, with a progressive specification, followed by attenuation within hours to days, depending on training state, exercise modality, intensity and other factors [5,6]. The acute stress imposed by a single exercise bout is in principle similar in both trained and untrained muscle when controlled for load, i.e. running to exhaustion. However, their transcriptional responses present intriguing differences. Disparities are apparent in both the types of genes that are activated or repressed, the time point of engagement, and the magnitude of modulation [5,[7], [8], [9]]. For example, genes associated with an inflammatory response are inhibited upon training, whereas oxidative respiration genes and different fiber type markers are activated [8]. Importantly, trained muscle also portrays a time shift towards faster induction and altered amplitudes [5]. Altogether, these observations suggest a progressive fine-tuning of transcription that helps to precisely control adaptations of muscle to exercise as opposed to a more general stress response in the first exercise bouts in an untrained muscle [6,10]. Specification of transcriptional signatures requires multi-layered epigenetic reorganization. Modifications of histones and DNA help to dictate chromatin structure and recruitment of regulatory proteins such as transcription factors, thereby by proxy affecting gene expression [11]. Changes in the muscular epigenetic landscape via DNA methylation can be rapidly made and reverted back to baseline, while others can be retained in muscle over weeks in acute and chronic exercise contexts [[12], [13], [14], [15]]. In addition, multiple histone modifications have been associated with training status [16,17]. So far, chromatin accessibility, a crucial rate-limiting step for transcription, has not been comprehensively studied in the context of exercise training. It is thus not understood to what degree muscle chromatin configurations are altered after exercise and if they differ depending on the training status. Furthermore, it is unknown if these changes persist beyond the acute post-exercise phase when transcription is reverted to baseline levels, and whether temporal shifts in chromatin remodeling are at play.

To elucidate the genome-wide temporal dynamics of chromatin accessibility for gene expression in the response to acute and chronic exercise, we utilized an assay for transposase-accessible chromatin combined with sequencing (ATAC-seq). These data were derived from muscle of endurance trained and untrained mice that were sacrificed in a temporal manner, specifically in the early (0 h and 6 h) and late phase (72 h) after an exercise bout. The heterogeneous make-up of skeletal muscle tissue, comprising various resident and infiltrating mononuclear cells besides multinucleated myofibers, imposes a level of complexity in understanding epigenetic regulation of the exercise-induced transcriptomic response [18]. To gain more specific insights, we therefore isolated myonuclei from the muscles of these mice, enabling the generation of myofiber-specific ATAC-seq data. We furthermore found a subset of genes in bulk RNAseq that is affected by differential chromatin accessibility and studied the expression of these genes to associate the transcriptional response post-exercise to altered chromatin conformations. Finally, we employed motif activity analysis to predict key transcription factor regulators on chromatin regions that underwent changes in accessibility. The combination of these approaches revealed new insights on the regulatory landscape of exercise and training adaptation.

## 2 Methods

### 2.1 Animals

C57/Bl6 wild-type (WT) mice were purchased from Janvier Labs. Mice were housed in an animal facility with a 12 h night/day cycle, with free access to food and water. Male mice were used at 22–26 weeks of age at sacrifice. All animal experiments were approved by the veterinary office of the canton Basel-Stadt in Switzerland, following federal, cantonal and institutional guidelines (Switzerland, Basel-Stadt and University of Basel, respectively).

### 2.2 Performance testing

Mice were acclimatized to the treadmill in anticipation of the maximal performance tests for 3 days, up to a maximal speed of 10 m/min within 20 min. Maximal performance was tested in a maximal capacity test on a treadmill at a 5° inclination. After 5 min at a starting speed of 8 m/min, the treadmill speed was increased by 2 m/min every 5 min, up to a maximal speed of 30 m/min, until mice reached exhaustion. VO2peak of a subset of mice was determined after 12 weeks of training using a short ramp protocol in which speed was increased by 0.03 m/min/s, as described in ref. [19].

### 2.3 Exercise training

At the age of 12 weeks, mice were randomly assigned to the training intervention group and placed in cages equipped with running wheels. Running wheel use (number of wheel turns) was recorded for the first 11 weeks of training. The endurance exercise training intervention lasted for 12 weeks total. Maximal performance tests were carried out at the end of these 12 weeks without a rest period. This last test was the acute bout of exercise after which trained and untrained control mice were sacrificed at 0 h, 6 h, or 72 h post-exercise.

### 2.4 Immunoblotting

*Quadriceps* muscles were crushed and homogenized in protein lysis buffer using Lysing Matrix S tubes (MP Biomedicals, #116921050-CF). Debris was removed by centrifugation. Protein was quantified using a Bradford assay and samples were diluted in Laemmli sample buffer, resolved in 4–20% precast mini gradient SDS-PAGE gels and transferred onto a Nitrocellulose membrane. Membranes were stained with Ponceau-S solution, blocked and incubated with a primary antibody that detects the electron transport chain subunits NDUFB8 (Complex I), SDHB (CII), UQCRC2 (CIII), MTCO1 (CIV), ATP5A (CV) (Abcam, #ab110413) and a secondary antibody (Dako, #P0260). eIF2 (Cell Signaling Technology, #9722) was used as a loading control. Antibody binding was detected using Pierce™ ECL chemiluminescence horseradish peroxidase (HRP) substrate detection kit (Thermo Scientific, #32106). Quantification was performed using FusionCapt Advanced v17.04.

### 2.5 Myonuclear isolation

Freshly prepared nuclei isolation media 1 and 2 (NIM1 and NIM2) and homogenization buffer (according to ref. [20]) were placed on ice. Nuclei were isolated from selected *quadriceps* muscles of 3 samples. All samples and buffers were kept on ice during the procedure. All steps were performed in the cold room. Muscles were removed from the −80 °C freezer and 150 mg of crushed tissue was transferred to Fastprep® 2 ml lysing matrix tubes containing a metal bead. 1 ml of freshly prepared homogenization buffer was added to the muscles, and the mixture was incubated on ice for 5 min. The samples were briefly vortexed and incubated another 5 min on ice. Subsequently, the tissue was homogenized using the MP Biomedicals™ FastPrep-24™ homogenizer, undergoing 2 cycles of 10-second shakes with a 1-minute break in between. After a further 5-minute incubation on ice, the homogenized samples were filtered through a Falcon® 100 um cell strainer into 50 ml tubes. Upon short spinning, the suspensions were filtered through Falcon® 50 um cell strainers into new 50 ml tubes. Tissue remnants on the strainers were rinsed with nuclei suspension buffer (1% BSA, 0.4 U/ul RNase inhibitor in DPBS). The 50 ml tubes were centrifuged at 700×*g* and 4 °C for 5 min. Pellets were resuspended in 1 ml of the suspension, which were transferred into pre-cooled 1.5 ml tubes by filtering throughs 30 um MACS® SmartStrainers. Rabbit anti-PCM1 antibody (1:200, Sigma Aldrich, HPA023370) was added to the samples and the mixtures were incubated for 45 min while slowly rotating. After centrifugation at 700×*g* and 4 °C for 5 min, the samples were washed once with LS Column buffer (0.5% bovine serum albumin, 2 mM EDTA, 1X RNasin® Plus Ribonuclease Inhibitor in DPBS). The pellets were resuspended in 80 ul of the suspension and subsequent magnetic labeling and separation of PCM1-labelled myonuclei was performed according to the manufacturer's protocol. In short, myonuclei were conjugated with anti-rabbit IgG microbeads (Miltenyi, #130-048-602). Samples were applied onto LS columns that were equilibrated with LS column buffer three times. Myonuclei were eluted into 15 ml tubes with 5 ml of LS column buffer after removing the column from the magnetic stand. The magnetic separation of myonuclei was repeated a second time and the final elutions were performed with 5 ml nuclei suspension buffer. After centrifugation at 750×*g* and 4 °C, the pellets were resuspended in 100 ul of supernatant. 1 ul of the samples were mixed with 9 ul of nuclei suspension buffer containing 1:1000 DAPI to determine nuclei count using a Neubauer chamber.

### 2.6 ATAC-sequencing and analysis

50′000 myonuclei were used to prepare ATAC-seq libraries, as previously described [21]. Briefly, nuclei were transposed using Tagment DNA TDE1 enzyme (Illumina, #20034197) at a final concentration of 145 nM in a buffer consisting of Tagment DNA buffer, digitonin, Tween-20, PBS and water as described [21]. DNA fragments were then barcoded using IDT® DNA/RNA UD Indexes (Illumina, #20027213), amplified and purified. DNA quality and quantity were assessed using Qubit fluorometer (Thermo Scientific) and Bioanalyzer 2100 (Agilent). Paired-end sequencing of the ATAC-seq libraries was performed using the Illumina NovaSeq 6000. Reads were trimmed using Trimmomatic version (v) 0.39 [22] using the parameters ILLUMINACLIP:/∼/NexteraPE-PE.fa:2:30:10:8:true MINLEN:60. Read quality was assessed using MultiQC v.1.11 [23] before and after trimming. Reads were mapped to the reference genome GRCm39 [24] and directly sorted by Bowtie v2.4.4 [25] and Samtools v1.15 [26], using the command bowtie2 and samtools sort with the parameters --very-sensitive -k 10 -p 8 –x. Peak calling was performed by Genrich v.0.6.1 (https://github.com/jsh58/Genrich) using the parameters -j -y -r -e chrM -v. Principal Component Analysis of normalized read counts for accessible regions was performed using DiffBind v.3.4.11 (https://bioconductor.org/packages/release/bioc/vignettes/DiffBind/inst/doc/DiffBind.pdf). Differential peak binding was computed using DESeq2-assisted analysis within the DiffBind package. Differential peaks were annotated using ChIPseeker v1.30.3 [27], utilizing proximity relation to nearest genes by the transcriptional start site (TSS), 5′ untranslated region (UTR), 3′UTR, exon, intron, downstream or intergenic regions, in that order of priority. Transcription factor activity networks were inferred using the software Cis-Regulatory Element Motif Activities (CREMA) (https://crema.unibas.ch/crumara/), which infers motif activity networks based on identified cis-regulator elements from chromatin accessibility data on the genome-wide scale. Chromatin accessibility changes were allocated based on changes, i.e. opening or closing of chromatin, in any of the exercised muscles compared to sedentary baseline control.

### 2.7 RNA isolation

Crushed *quadriceps* muscle tissue was further homogenized using Lysing Matrix D tubes (MP Biomedicals 6913-500). Muscle homogenates collected in TRI Reagent (Sigma–Aldrich, #T9424), and RNA was extracted and purified using the RNeasy kit (Qiagen, #74104). RNA quality was assessed using Bioanalyzer 2100 (Agilent).

### 2.8 RNA-sequencing and analysis

RNA-seq libraries were prepared using a TruSeq RNA Library Prep Kit version (v) 2 (Illumina, #RS-122-2001). Paired-end sequencing was performed using the Illumina NovaSeq 6000. Reads were mapped to reference genome GRCm39 using STAR [28]. Counts were quantified using salmon v 1.5.0 [29] and transcript per million (TPM) values were collected to form a count matrix. The gene expression data obtained from bulk muscle was subsequently stratified using the ATAC-seq results. The latter were obtained from myonuclei based on PCM1-based sorting, and are devoid of marker genes for mononuclear cells (Suppl. Fig. S4a). Thus, a list of myonuclear genes with ATAC-seq peaks was compiled, further stratified by differential accessibility in any of the conditions and time points. By limiting the RNA-seq analysis to genes that overlap with the myonuclear ATAC-seq-derived gene list, we deconvolved transcriptional changes observed in RNA-seq into myonuclear and mononuclear origins.

### 2.9 Gene Ontology analysis

Gene Ontology (GO) analysis was performed on ATAC-seq, RNA-seq and proteomic data using the Database for Annotation, Visualization and Integrated Discovery v6.9 (DAVID; https://david.ncifcrf.gov/) [30,31]. To accommodate the prediction of chromatin accessibility (from the ATAC-seq) to gene annotation, no background lists were used.

### 2.10 Transcription factor activity

Activity of transcription factors was analyzed for ATAC-seq data using Cis-Regulatory Element Motif Activities (CREMA; https://crema.unibas.ch/crumara/).

### 2.11 Statistics

Statistical significance of *in vivo* data was determined using GraphPad Prism v8.0 using unpaired two-tailed t-tests, indicated in figure legends accordingly. These were applied after confirming data normality using QQ-plots. P values < 0.05 were considered significant. Values are expressed as mean ± SD. Differential accessibility was calculated by comparing peaks of all conditions to sedentary. A significance cutoff of FDR<0.05 was applied.

## 3 Results

### 3.1 Endurance training amplifies rapid exercise-induced chromatin closing

Mice were endurance trained with continuous access to voluntary running wheels throughout the training period (Suppl. Fig. 1a). Trained mice showed enhanced performance and VO_2_ peak in maximal capacity tests when compared to untrained mice (Suppl. Fig. 1b–c). Muscles of trained mice portrayed muscular adaptations characteristic of this type of training [32], including *soleus* hypertrophy and increased expression of electron transport chain subunits (Suppl. Fig. 1d–f).

**Figure 1:**
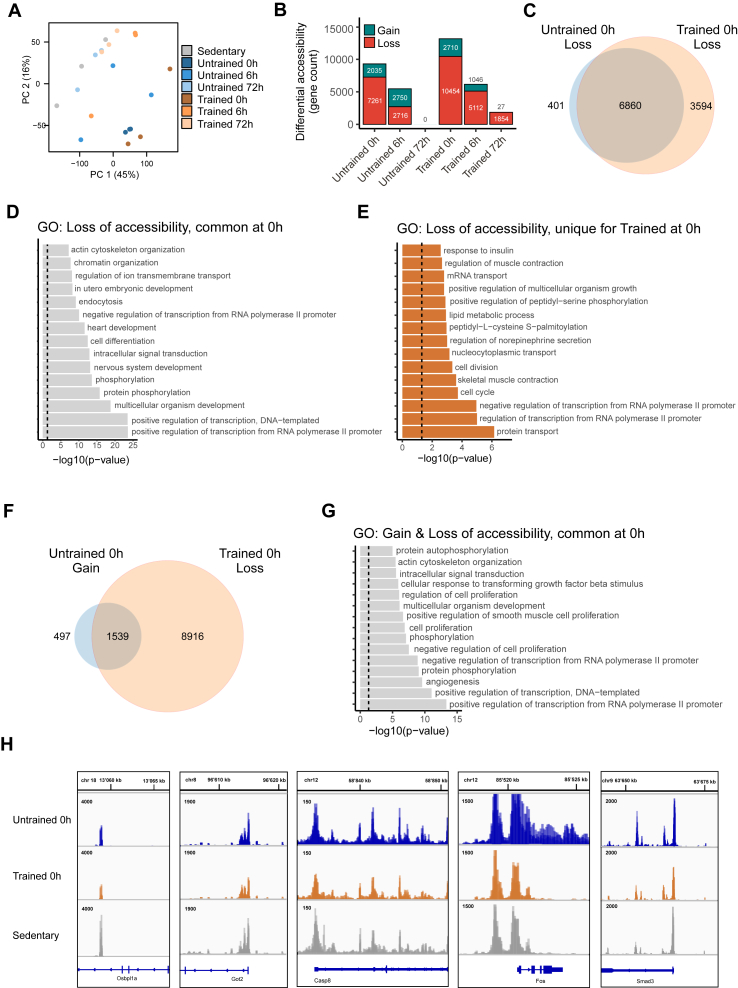
**Endurance training enhances rapid exercise-induced chromatin closing.** (**A**) PC1 and 2 coordinates of normalized read counts for accessible regions from ATAC-seq. (**B**) Differential accessibility events, divided by gain or loss of accessibility, associated to gene names by closest transcriptional start site (**C**) Venn diagram of genes associated to regions that lost accessibility at time point 0 h, compared to sedentary. (**D**) Top 15 Gene Ontology (GO) biological process (BP) terms, enriched in genes with chromatin accessibility loss at 0 h, common for untrained and trained conditions. (**E**) Top 15 GO BP terms, enriched in genes unique to trained 0 h in chromatin accessibility loss. (**F**) Venn diagram of genes associated to gained accessibility in untrained 0 h, and lost accessibility in trained 0 h. (**G**) Top 15 GO BP terms enriched in genes of the overlap between untrained 0 h-gained and trained 0 h-loss of chromatin accessibility. (**H**) Representative genome browser view of chromatin accessibility peaks of genes that show differential accessibility at 0 h post-exercise. *Osbpl1a*: similar loss in trained and untrained muscle; *Got2*, *Casp8*: loss primarily in the trained state. *Fos*, *Smad3*: gain in the untrained, loss in the trained state. In 1 A, B, C, F: Differential accessibility (loss & gain): every condition vs. sedentary, FDR<0.05. In 1D, E, G: Dashed line indicates a significance threshold of P = 0.05.

Chromatin accessibility landscapes of myonuclei, isolated from sedentary, as well as trained and untrained mice sacrificed at 0 h, 6 h and 72 h after an acute bout of exhaustive treadmill running were mapped using ATAC-sequencing (ATAC-seq). The purity of the myonuclear fractions used for ATAC-seq is evident by the absence of sequencing reads forming peaks in marker genes of mononucleated cells that are found in bulk muscle tissue of sedentary mice (Suppl. Fig. 2a). Principal component (PC) analysis revealed no clear separation of samples along the PC1 and PC2 axes, even though the 0 h samples cluster in the lower right, and the 72 h and sedentary samples in the upper left quadrant, hinting at a multicomponent contribution to the separation of early events at 0 h from the rest (Figure 1A). We associated the differentially accessible regions, based on the comparison between baseline sedentary and the exercised samples, to genes through proximity relation to the closest genomic region, prioritizing the TSS. We then stratified the regions that were associated to genes according to the direction of change in chromatin accessibility (gain or loss) compared to sedentary, baseline control muscle. Chromatin accessibility changes (compared to the sedentary state) were prominent in distal regulatory regions, including intergenic regions and introns (∼76.8%) and in promoters (∼10.5%) (Suppl. Fig. 2b).

**Figure 2:**
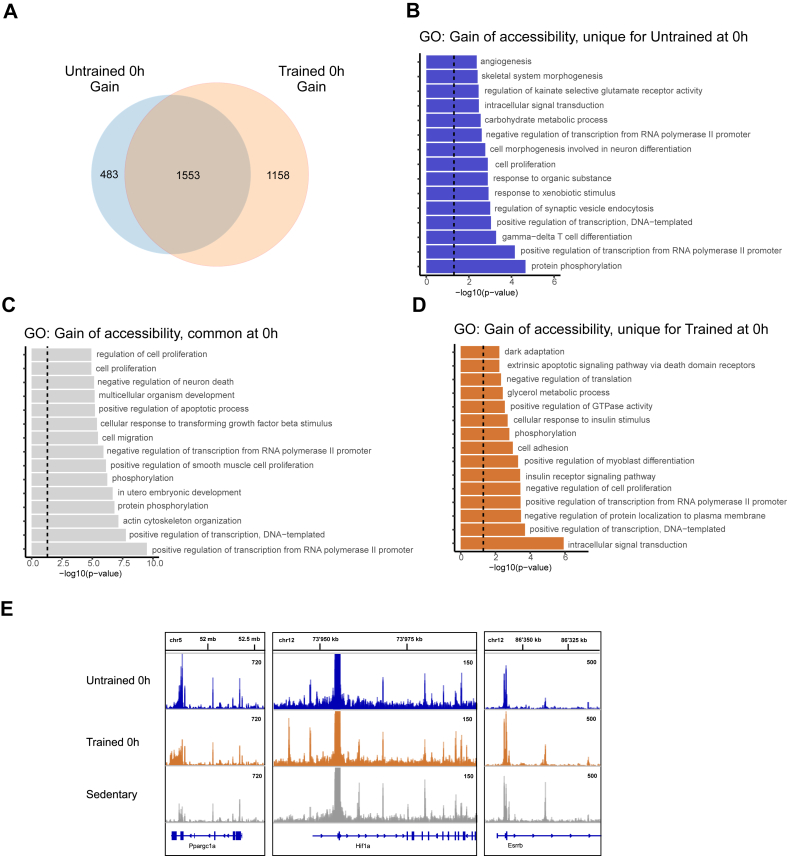
**Gain of chromatin accessibility contributes to a training-specific post-exercise response.** (**A**) Venn diagram of genes associated to regions that gained accessibility at time point 0 h, compared to sedentary. (**B**) Top 15 Gene Ontology (GO) biological process (BP) terms, enriched in genes with chromatin accessibility gained at 0 h, unique for the untrained condition. (**C**) Top 15 GO BP terms, enriched in genes with chromatin accessibility gain at 0 h, common for untrained and trained conditions. (**D**) Top 15 GO BP terms, enriched in genes unique to trained 0 h in chromatin accessibility gain. (**E**) Representative genome browser view of chromatin accessibility peaks of genes that show differential accessibility. *Ppargc1a*, *Hif1a*, *Esrrb*: gain and loss of chromatin accessibility in different intragenic regions. In 2 A: Differential accessibility (gain): every condition vs. sedentary, FDR<0.05. In 2 B, C, D: Dashed line indicates a significance threshold of P = 0.05.

**Figure 3:**
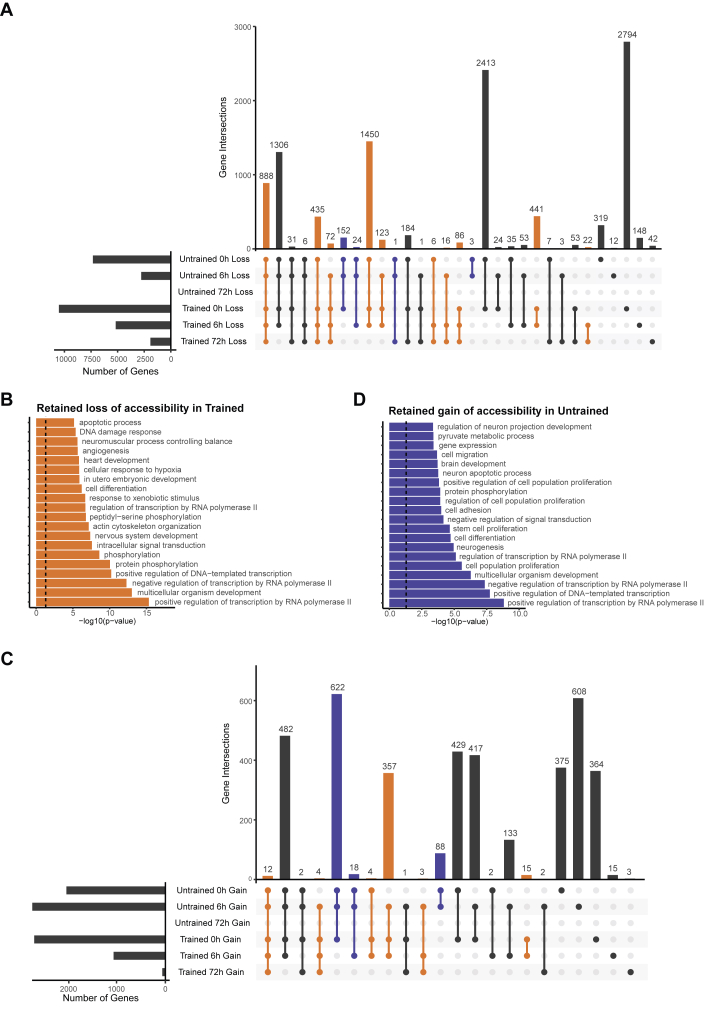
**Training state-linked chromatin accessibility changes are robust and retained beyond the acute exercise phase.** (**A**) Intersections of genes associated to regions that lost chromatin accessibility. Intersection colors indicate retention of genes across two or more time points, with larger retention in the trained condition (orange) or untrained condition (blue). (**B**) Top 15 Gene Ontology (GO) biological process (BP) terms, enriched in genes with chromatin accessibility loss that is retained in the trained condition (sum of orange in panel A). (**C**) Set intersections of genes associated to regions that gained chromatin accessibility. Intersection colors indicate retention of genes across two or more time points, with larger retention in the trained condition (orange) or untrained condition (blue). (**D**) Top 15 GO BP terms, enriched in genes with chromatin accessibility gain retained in the untrained condition (sum of blue subsets in panel C). In 3 B, D: Dashed line indicates a significance threshold of P = 0.05.

This analysis revealed a notable overrepresentation of chromatin accessibility loss acutely after exercise, with most accessibility lost at time point 0 h for untrained and trained muscle. Chromatin closure in untrained muscle progressively declined at 6 h to complete absence at 72 h (Figure 1B). In contrast, gain of accessibility was greater at 6 h compared to 0 h, but also gone at 72 h in untrained muscle. Endurance training amplified the loss of chromatin accessibility at all time points, while retaining the temporal pattern with a peak at 0 h. The majority of the accessibility loss found in the untrained condition at 0 h was shared with the trained condition (Figure 1C). Overall, a general attenuation of change in accessibility in both training states is indicated by the large overlap between the regions in all three time points, indicated by the small numbers of regions that are exclusively observed at later time points (Suppl. Fig. 2c–f). A notable exception is found in gain-of-accessibility in the untrained muscle in which a majority of number of regions (∼55%) arose *de novo* at the 6 h time point (Suppl. Fig. 2c–f). Gene ontology (GO) analysis of genes that undergo common loss of accessibility showed enrichment in multiple terms that reflect the acute exercise phase, namely chromatin and transcriptional regulation, phosphorylation and signal transduction (Figure 1D). Genes associated to loss of accessibility that is unique to trained muscle showed enrichment in metabolic terms such as lipid metabolic process and response to insulin (Figure 1E). Unique for untrained muscle were enriched terms associated to the immune system (Suppl. Fig. 3a). Given that the number of genes with gained chromatin accessibility in untrained 0 h (Figure 1B and 2′035 genes) has a proportional similarity with the excess loss of accessibility observed in trained 0 h when compared with untrained 0 h (Figure 1B and 3′193 genes based on the difference between 10′454 of the trained and 7′261 genes of the untrained at 0 h), we hypothesized that this may reflect an overlap of genes showing opposite accessibility changes in response to training. Indeed, 76% of the genes associated to gained accessibility in untrained muscle at 0 h post-exercise undergo closing in its trained counterpart (Figure 1F). Terms associated to this overlap include transcriptional regulation, angiogenesis and growth (Figure 1G). However, these genes only contribute to around 15% of all of the genes with loss of accessibility in the trained muscle at 0 h, indicating a much larger specification in the trained muscle beyond the reversal from regions with gain-of-function in the untrained muscle (Figure 1F). The opposite direction, gain of accessibility in trained with loss of accessibility in untrained muscle at 0 h also yielded significant overlap (Suppl. Fig. 3b). Of note are terms intracellular signal transduction and nervous system development (Suppl. Fig. 3c). At time point 6 h, we observe a similar pattern as in 0 h of overrepresentation of chromatin closing in the trained condition (Figure 1B). Of note, 51% of the accessibility gained in untrained muscle at 6 h post-exercise corresponds to genes that undergo chromatin closing in trained muscle (Suppl. Fig. 3d). The genes that contribute to this overlap enrich in GO terms, some of which are the same as observed in the 0 h post-exercise comparison (Figure 2B,C), including positive regulation of transcription from RNA polymerase II promoter, positive regulation of transcription - DNA templated, angiogenesis, protein phosphorylation or multicellular organism development (Suppl. Fig. 3e). Examples of different patterns of chromatin accessibility changes are depicted (Figure 1H). Altogether, these signatures, which exhibit both shared and distinct patterns of change in the acute phase post-exercise, along with their functional annotations, suggest a training-induced specification of the chromatin accessibility landscape.

### 3.2 Gain of chromatin accessibility contributes to training-specific post-exercise response

To further investigate post-exercise chromatin remodeling and its role in the acute phase, associated with prominent gene induction, we focused on the gain of chromatin accessibility at the 0 h time point. Genes associated with gain of accessibility showed slightly smaller overlaps (49%) between trained and untrained muscle (Figure 2A) compared to loss of accessibility (63%) (Figure 1C). Unique gains of accessibility in the untrained condition reflect immune, metabolic and neuron-related processes (Figure 2B). Genes that undergo gain of accessibility in both untrained and trained muscle revealed terms related to phosphorylation, growth and apoptosis (Figure 2C). Terms enriched for genes with gained accessibility in the trained condition only included signal transduction and insulin signaling (Figure 2D). Both shared and unique gains of accessibility for trained and untrained muscles exhibit similar GO terms as found for accessibility loss, for example regulation of transcription. Per gene, multiple peaks that indicate changes in accessibility are often found, which however can be in opposite directions (Figure 2E). This observation underlines the complexity of gene regulation through chromatin remodeling. Chromatin accessibility results therefore can only meaningfully be interpreted together with gene expression data. Furthermore, prediction or experimental validation of regulatory factor recruitment to this region, e.g. that of transcription factors activating or inhibiting gene expression, help to further analyze this type of data.

### 3.3 Chromatin accessibility changes are robust and retained beyond the acute exercise phase due to training

Next, we explored whether accessibility changes are retained over time, by comparing gene overlaps between time points 0 h, 6 h and 72 h post-exercise in the untrained and trained conditions. When focusing on the subsets of genes marked by loss of accessibility, we observed substantial overlaps between multiple time points within the untrained or trained condition, which shows retention of this loss over time (Figure 3A). Here, we assigned colors by selecting unique retention, or longer retention within one condition (orange for trained, blue for untrained) to address the effect of training. The number of genes that show longer retention across two or three time points is higher in trained muscle, which is not matched in the untrained condition. This is in part explained by the absence of loss of accessibility at time point 72 h in untrained muscle. Strikingly, the majority of genes that show loss of chromatin accessibility in the 72 h time point for trained muscle is shared with other time points. This reflects a robust effect of changes acquired at earlier time points. The GO terms associated with these genes that retain more loss of chromatin accessibility in the trained condition, or for longer (orange), again show terms that match training adaptations, for example nervous system development, hypoxia response and angiogenesis (Figure 3B). Analyzing the gene overlaps across time in gain of accessibility yielded more retention in the untrained condition (blue) (Figure 3C). These genes were enriched for more GO terms associated with the nervous system and metabolism (Figure 3D). Overall, these findings suggest that chromatin accessibility changes persist over time in a robust manner. Training enhances the stability of the alterations with regard to loss of accessibility in particular, up until 72 h post-exercise. Finally, the highest number of chromatin accessibility changes associated with genes are found at 0 h: intriguingly, most of these occur uniquely in the trained state (2′794 genes/regions), indicating a high degree of specification, followed by 2′413 genes with associated chromatin regions that are shared between the 0 h time points in untrained and trained muscle marked by loss of accessibility (Figure 3A). The subsequent groups include 1′450 genes/regions with a higher retention in the trained state (0 h in untrained, 0 h and 6 h in trained), 1′306 genes/regions with unaltered kinetics (found in both untrained and trained 0 h and 6 h time points), and 888 genes/regions with higher retention in the trained muscle (0 h and 6 h in the untrained, 0 h, 6 h and 72 h in the trained).

### 3.4 Exercise-induced, myonuclei specific genes associated to chromatin accessibility changes show varying expression patterns

**Figure 4:**
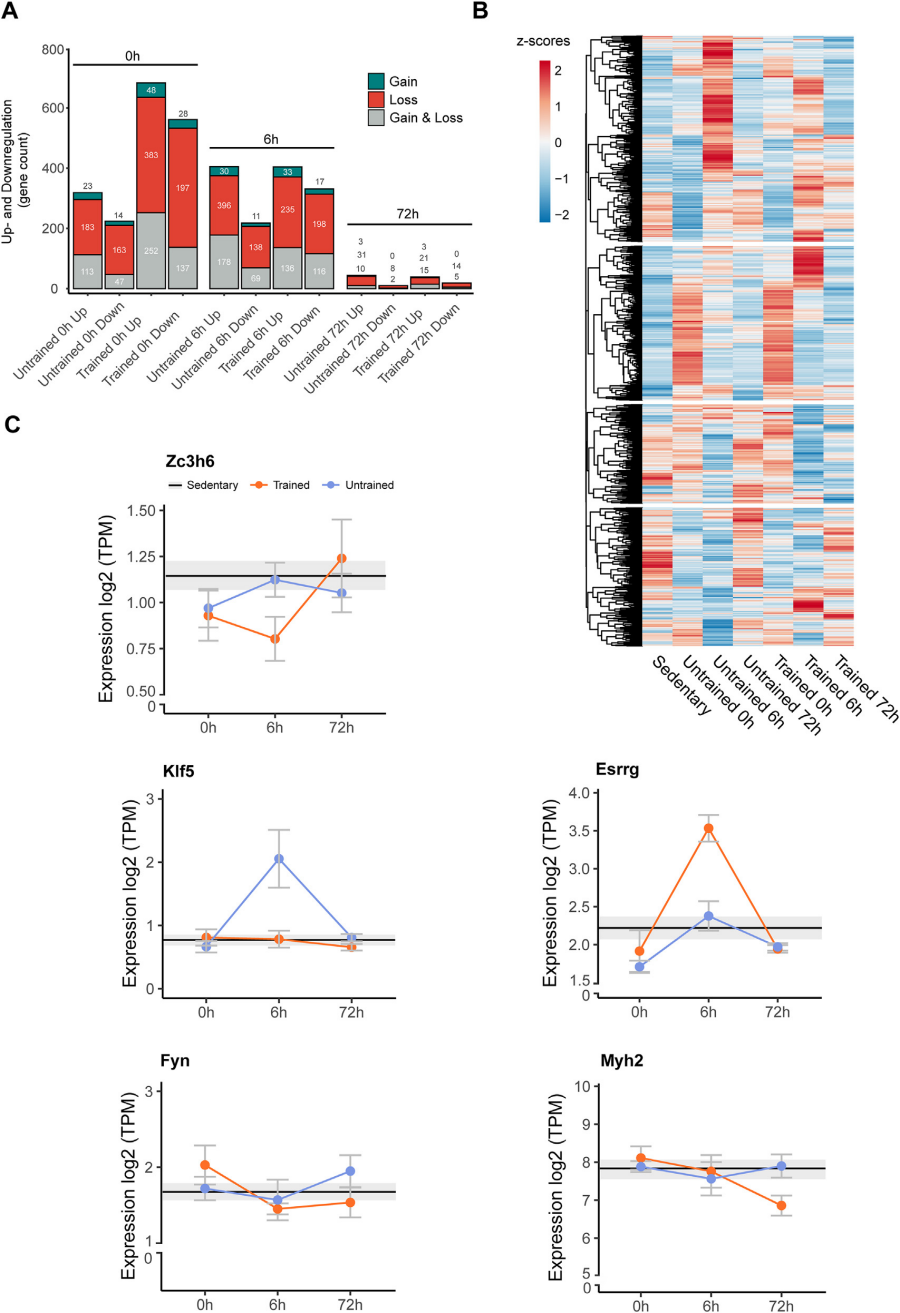
**Exercise-induced, myonuclei-specific genes associated to chromatin accessibility changes show different expression patterns.** (**A**) Number of genes significantly up- or downregulated (log2 fold change ± 0.585, FDR <0.05) and their association to gain, loss or gain and loss of chromatin accessibility of myonuclei-specific genes from Suppl. Fig. 4a. (**B**) Heatmap of z-scores [calculated as mean (log2 (TPM))] from gene expression data of genes associated with chromatin accessibility changes in ATAC-seq data, that show significant up- or downregulation (log2 fold change ± 0.585, FDR <0.05) in one or more conditions versus sedentary, clustered based on Euclidean distance. (**C**) Gene expression levels of genes (in Log2 TPM) present in subset of genes described in A-B.

Next, we wanted to assess the impact of chromatin accessibility changes on the transcriptomic level. Thus, to extrapolate the ATAC-seq findings in myonuclei to bulk RNA-seq data, we compiled a subset of genes whose regulation was associated with differential chromatin accessibility found in the myonuclear fraction in any of the exercise and training conditions compared to sedentary controls. Thus, a list of genes that most likely are myonuclear-specific was derived from the ATAC-seq data, which subsequently was compared to the RNA-seq results obtained from bulk muscle. Thereby, a prediction for the stratification of bulk sequencing data into myonuclear and mononuclear cell gene expression was achieved. The myonuclear subset accounts for 23% of all genes identified in bulk RNA-seq data from muscle tissue (Suppl. Fig. 4a). The majority of these genes were associated to loss of chromatin accessibility, while also a substantial number of genes was associated to both gain and loss. This is due to the presence of chromatin accessibility changes in multiple genomic regions associated to the same gene. We then discerned how many of the myonuclei-specific, exercise induced transcripts show significant up- or downregulation in the different conditions, and whether this was associated to chromatin accessibility loss, gain or either of the two within the same gene. At time point 0 h, we found a higher number of up- and downregulated genes associated mainly to loss of accessibility in trained muscle, when comparing to the untrained muscle (Figure 4A, Suppl. Fig. 5). This transcriptional difference due to training status was partially dampened at 6 h post-exercise, with similar numbers between upregulated genes in trained and untrained conditions, while the number of downregulated genes remained higher in the trained compared to the untrained data. Finally, very few transcriptomic changes were seen 72 h post-exercise in either condition, which is in concordance with bulk muscle data post-exercise published previously [5]. We then plotted the 1′848 genes that show up- or downregulation in at least one condition as z scores calculated from gene expression counts. This revealed various gene expression patterns, ranging from increased and decreased expression at different time points for untrained and trained muscles (Figure 4B). Some patterns are illustrated in the depiction of individual genes with an exercise response in a temporal and/or training-state-dependent manner (Figure 4C). For example, the expression of the transcription factors Klf5 [33] and Essrg [34], both well-described exercise responsive genes, show a strong training-state dependency (Figure 4C). Functionally, very similar GO terms emerged (Suppl. Fig. 4b) as in the previous analysis of the ATAC-seq data of all chromatin regions with changes in accessibility (Figures 1D–E, G, 2C–D, 3B, D, S3a, S3c). Moreover, it is clear that directionality of transcriptional change, i.e. up- or downregulation, is only loosely associated with gain- or loss of accessibility of chromatin, hinting at a complex regulatory mechanism.

### 3.5 Repression of immediate early transcription factors alters the transcriptional response post-exercise

**Figure 5:**
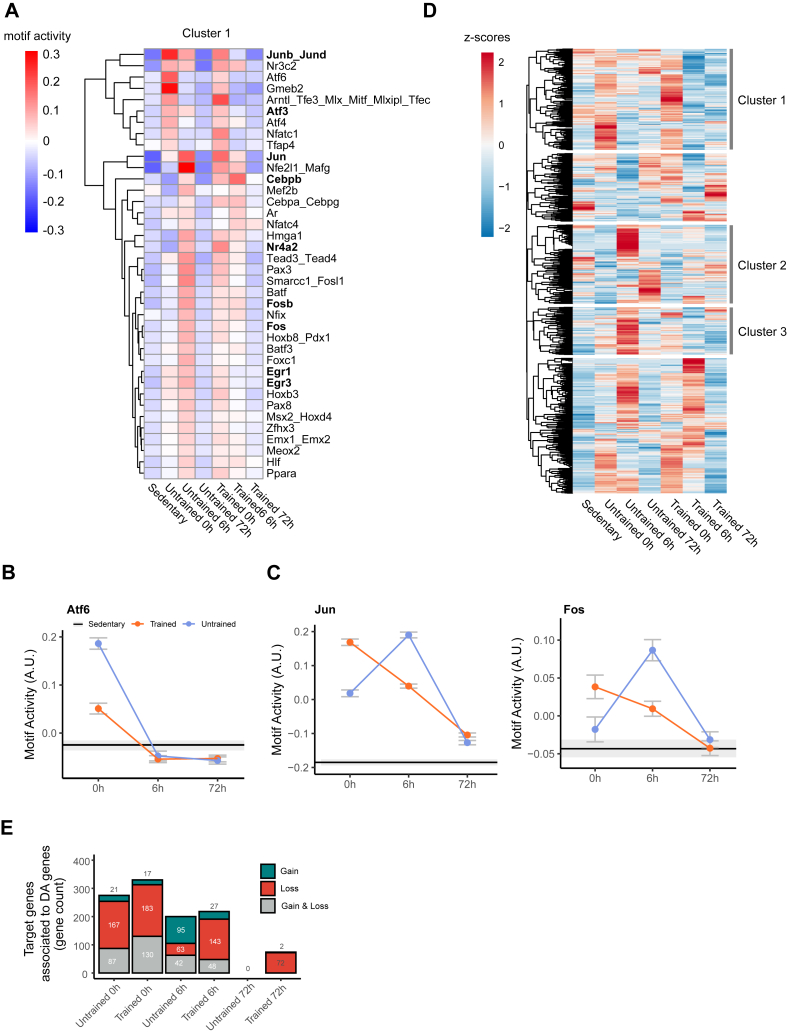
**Repression of immediate early transcription factor motif activities alters the transcriptional response post-exercise.** (**A**) Heatmap of motif activity (A.U) from ATAC-seq data, data was clustered based on Euclidean distances. Motifs for immediate early genes are marked in bold. (**B**–**C**) Motif activity (A.U.) plotted for motifs present in panel A. (**D**) Heatmap of z-scores [calculated as mean (log2 (TPM))] from gene expression data of the predicted top 200 target genes from motifs of interest (marked in bold in panel A), clustering based on Euclidean distances. (**E**) Number of target genes of immediate early gene motifs, extracted from clusters 1, 2, 3 in panel D, associated to gain or loss, or gain and loss of chromatin accessibility.

To dissect the mechanism of training-induced differences in the transcriptional response to exercise, we performed Cis-Regulatory Element Motif Activities (CREMA) analysis on the chromatin accessibility changes. Among all detected motif activities, a subset of motifs (Cluster 1) displayed the most substantial training-dependent differences between the early post-exercise time points (0 h and 6 h), as compared to 72 h and the sedentary state (Suppl. Fig. 6). Even though a strong overlap in motifs between untrained and trained samples exist in this cluster, the temporal dynamics are often different. A few motifs are predicted to be strongly activated primarily in untrained muscle at 0 h, for example Atf6, while others, e.g. Jun, Fos or other immediate early genes such as Junb_Jund, Atf3, Cebpb, Nr4a2, Egr1 and Egr3, show an opposite pattern (Figure 5A–C). In general, many motifs are predicted to be activated in trained muscle in a more blunted and dispersed manner, thus both at 0 h and 6 h, while the same motifs clearly peak at 6 h in the untrained myonuclei, thereby exhibiting a delayed response. The transcription factors with significantly predicted changes in activity are involved in the regulation of many different cellular functions. As proof-of-concept, and based on the enrichment of transcription factors contributing to the early and intermediated stress response (marked in bold in Figure 5A) [35], we investigated this process in more detail. This pathway comprises a number of signaling and transcriptional events linked to a generic stress response upon various perturbations in different cell types [35]. Of note, even though this pathway is prominently engaged in acute exercise paradigms [6,10], the functional consequence is still poorly understood [33]. Thus, to assess the impact of these genes in myonuclei-specific exercise-induced gene regulation, we created a subset of the top 200 target genes of the motifs of these immediate early genes (1′221 in total), and plotted their transcriptional expression as z-scores. This showed distinct expression patterns, namely more pronounced upregulation at 0 h, followed by downregulation in later time points particularly in trained muscle (cluster 1), and upregulation at 6 h or 72 h in the untrained state (cluster 2 and 3) (Figure 5D). The chromatin accessibility changes present in these target genes that follow this pattern mirror this, showing a larger number of gained accessibility in the untrained 6 h condition, versus more loss of accessibility in its trained counterpart (Figure 5E). Altogether, this suggests a training-dependent mitigation of the immediate early stress response that is linked to the chromatin landscape.

## 4 Discussion

Exercise-induced muscular adaptations are governed by multi-layered regulation. The substantial difference in the response of an untrained and a trained muscle to an acute exercise bout suggests the involvement of differential epigenetic regulation [5]. We now demonstrate that remodeling of chromatin accessibility could contribute to this training state-dependent process. In this study, we observed a surprising dominance of rapid loss of chromatin accessibility in muscle myonuclei despite an inverse distribution of up- and downregulated genes. The overrepresentation of loss of chromatin accessibility in our data is consistent with the finding that endurance training causes hypoacetylation of enhancers [17]. It is currently unclear how these changes are brought about, but prior studies have shown that the formation of heterochromatin and gene silencing can be induced by thermal and mechanical stress [[36], [37], [38]] - stimuli that are also implicated in the response of muscle to exercise [3]. It is likewise unknown to what degree past exposure to such perturbations can enhance these effects and prepare myofibers for future stress conditions.

Overall, no clear correlation between gain and loss of chromatin accessibility and the directionality of changes in gene expression emerged (Suppl. Fig. 5). This can be interpreted in various manners. First, changes in accessibility status were defined by specific statistical thresholds, which could preclude more precise comparisons of differences in peak amplitude. Moreover, we did not investigate how peak width, and hence coverage of genomic regions, was altered. Such changes could entail the ex- or inclusion of regulatory motifs for transcription factors and thereby also affect gene expression. Furthermore, transcriptional downregulation may depend much more on dynamic changes in chromatin accessibility than upregulation. Obviously, a temporal disconnect between changes in chromatin accessibility and gene transcription might exist, which, despite covering three time points in our study (0 h, 6 h, 72 h), might not be represented in our results due to temporal shifts between changes in chromatin accessibility and transcriptional regulation or transient (or non-productive) states that are incompletely recorded in the time points that were studied. Importantly, a direct correlation would only be expected in a subset of associations. For example, open chromatin can be permissive, poised or linked to an enhancer-promoter disconnect, in which case transcriptional control would only occur in specific contexts [39]. Moreover, increased chromatin accessibility can also accommodate the binding of transcriptional repressors, thereby confining gene expression [40]. In addition, various factors, including nucleotide positioning, DNA methylation, histone modifications, non-coding RNAs, cis-regulatory elements or the three-dimensional chromatin structure affect this process [41]. Thus, seemingly discordance between chromatin accessibility and transcriptional activity occurs, e.g. described in ref. [42], indicating that opening and closing of chromatin is permissive, but not strictly deterministic for gene expression changes. Nevertheless, for a subset of genes in our data, a better correlation exists. For example, loss of accessibility in trained muscle effectively suppressed the immediate early and intermediate stress response to exercise, affecting the predicted activity of various immediate early transcription factors, which will have to be experimentally confirmed. The activation of these factors, for example Fos and Jun, and the involvement in shaping the post-exercise transcriptional response in particular in untrained muscle has been reported previously [43,44]. We now provide an explanation for the temporal shift towards faster activation and attenuation in trained muscle based on the concomitant chromatin accessibility changes in this context. Accordingly, this could be linked to the shift in gene expression kinetics to earlier time points, and the larger proportion of transcriptional downregulation as previously reported in endurance trained muscle [5]. It is also conceivable that inverse relationships exist, i.e. gene expression changes, such as the induction of the peroxisome proliferator-activated receptor γ coactivator 1α (PGC-1α) leading to alterations in chromatin accessibility, in the case of PGC-1α by the recruitment of histone acetyltransferase, Mediator and Swi/Snf complexes to different regulatory regions [45]. A more fine-grained temporal analysis as well as targeted gain- and loss-of-function experiments could help to further elucidate such aspects in future studies. Intriguingly, some of the regulation exceeds a mere attenuation in a training state-dependent manner. For example, genes associated to regions with higher accessibility in the untrained muscle were found that underwent active closing in the trained samples, demonstrating context-dependent active remodeling. Moreover, more long-term effects were observed in the trained samples, persisting at 72 h post-exercise, a time point at which only very few genes still show altered transcription. Thus, chronic alterations in chromatin accessibility could be linked to corresponding epigenetic changes that prime trained muscle for a differential response to an acute exercise bout compared to untrained muscle. Therefore, the existence of closed chromatin regions at 72 h post-exercise in trained muscle could contribute to the concept of muscle memory, which proposes that long-lasting changes elicited by training persist periods of detraining, at least for a certain time, and subsequently facilitate training adaptations once exercise is resumed [13]. Chromatin accessibility could be linked to DNA methylation changes, which were observed at 24 h post-exercise in humans, and upon retraining, and thus have been associated with muscle memory [[13], [14], [15]]. We concede that our observations of retention of chromatin accessibility are limited to 72 h post-exercise, and therefore, to make stronger conclusions about muscle memory, longer time points will have to be assessed in future studies, optimally in different training paradigms and contexts. For example, resistance training-induced changes to the DNA methylome in human muscle are largely lost after 7 weeks of detraining [13]. In other modalities, e.g. high intensity interval training, methylation changes were retained even after 3 months [46]. Other factors such as diet [47] or disease [48] further modulate the skeletal muscle methylome in exercise.

In resistance exercise, DNA methylation signatures differ significantly between myonuclei and mononuclear cell nuclei [49]. In this study, the isolation of myonuclei from bulk tissue refined the mapping of muscle enhancers [18]. In a similar approach, we leveraged myonuclear ATAC-seq data on chromatin accessibility to gain novel insights into gene regulation in muscle fibers responding to acute endurance exercise bouts in a training state-dependent manner. Hopefully, future studies will aim at performing such experiments at the single nucleus level, enabling the analysis of individual myonuclear populations, integrating chromatin accessibility, DNA methylation, histone modifications and gene expression data. However, even at the level provided here, novel insights into the regulatory mechanisms and landscape of exercise adaptation are provided. These are crucial to understand how training adaptation is brought about, which is associated with powerful health benefits in a large number of different diseases [2], and projects these beyond muscle tissue [[50], [51], [52]]. Thereby, potential novel targets for the prevention and/or treatment of these pathologies could be identified.

## CRediT authorship contribution statement

**Laura M. de Smalen:** Writing – review & editing, Writing – original draft, Visualization, Validation, Methodology, Investigation, Formal analysis, Conceptualization. **Volkan Adak:** Validation, Methodology, Investigation, Formal analysis. **Aurel B. Leuchtmann:** Validation, Methodology, Investigation, Formal analysis. **Konstantin Schneider-Heieck:** Validation, Methodology, Investigation, Formal analysis. **Stefan A. Steurer:** Methodology, Investigation, Formal analysis. **Christoph Handschin:** Writing – review & editing, Writing – original draft, Validation, Supervision, Project administration, Funding acquisition, Formal analysis, Conceptualization.

## Declaration of competing interest

The authors declare the following financial interests/personal relationships which may be considered as potential competing interests: Christoph Handschin reports financial support was provided by Swiss National Science Foundation. If there are other authors, they declare that they have no known competing financial interests or personal relationships that could have appeared to influence the work reported in this paper.

## Data Availability

ATAC-seq data have been deposited in Gene Expression Omnibus (GEO) under accession number GSE302910. RNA-seq data have been deposited in GEO under accession number GSE302911. Other data will be made available on request.

## Appendix A Supplementary data

Multimedia component 1 Supplementary Figure 1. **Adaptations induced by voluntary running wheel endurance training.** (**A**) Number of voluntary running wheel turns in 24 h in trained animals. (**B**) Distance covered in maximal capacity test in meters. (**C**) VO2peak reached in a maximal capacity test. (**D**) Absolute mass for quadriceps (Quad) and soleus muscles of trained and untrained mice, averaged across both limbs. (**E**) Protein expression of electron transport chain subunits in quadriceps muscle. (**F**) Quantification of electron transport chain subunits expression, relative to loading control eIF2. In 1B-D: unpaired two-tailed t-test ∗p < 0.05, ∗∗∗<0.001 describe the training effect. Multimedia component 1

Multimedia component 2 Supplemental Figure 2. **Localization and differences in chromatin accessibility across conditions.** (**A**) Distribution of differential chromatin accessibility across the genomic regions. The results depicted from sedentary animals is representative of similar data in the other experimental cohorts. (**B**) Pie chart representing the average distribution of genomic regions of differential chromatin accessibility across all conditions. (**C**) Venn diagram of genes associated to lost accessibility in untrained 0h and 6h. (**D**) Venn diagram of genes associated to gained accessibility in untrained 0h and 6h. (**E**) Venn diagram of genes associated to lost accessibility in trained 0h, 6h and 72h. (**F**) Venn diagram of genes associated to gained accessibility in trained 0h, 6h and 72h. Multimedia component 2

Multimedia component 3 Supplemental Figure 3. **Characterization of chromatin accessibility changes** (**A**) Top 15 Gene Ontology (GO) biological process (BP) terms, enriched in genes chromatin accessibility loss at 0h, only in untrained. (**B**) Venn diagram of genes associated to lost accessibility in untrained 0h, and gained accessibility in trained 0h. (**C**) Top 15 GO BP terms enriched in genes of the overlap between untrained 0h-lost and trained 0h-gain of chromatin accessibility. (**D**) Venn diagram of genes associated to gained accessibility in untrained 6h, and lost accessibility in trained 6h. (**E**) Top 15 GO BP terms enriched in genes of the overlap between untrained 6h-gained and trained 6h-loss of chromatin accessibility. In S2C: Differential accessibility (loss & gain): every condition vs. sedentary, FDR<0.05. In S2B, D: Dashed line indicates a significance threshold of P = 0.05. Multimedia component 3

Multimedia component 4 Supplemental Figure 4. **Proportions and functional annotation of transcripts associated to myonuclei-specific, exercise-induced genes from ATAC-seq data.** (**A**) Pie chart representing the proportions of myonuclei (non-)specific genes from bulk RNA-seq count matrix, and the occurrence of these genes in gain and loss of accessibility list from ATAC-seq. Differential accessibility (loss & gain): every condition vs. sedentary, FDR<0.05. Multimedia component 4

Multimedia component 5 Supplementary Figure 5. **Correlation between chromatin opening/closing and gene expression changes.** (**A**) Heatmap of z-scores [calculated as mean (log2 (TPM))] from gene expression data of genes associated with gain of chromatin accessibility in ATAC-seq data, that show significant up- or downregulation (log2 fold change ± 0.585, FDR <0.05) in one or more conditions versus sedentary, clustered based on Euclidean distance. (**B**) Heatmap of z-scores [calculated as mean (log2 (TPM))] from gene expression data of genes associated with loss of chromatin accessibility in ATAC-seq data, that show significant up- or downregulation (log2 fold change ± 0.585, FDR <0.05) in one or more conditions versus sedentary, clustered based on Euclidean distance. (**C**) Heatmap of z-scores [calculated as mean (log2 (TPM))] from gene expression data of genes associated with gain and loss of chromatin accessibility in ATAC-seq data, that show significant up- or downregulation (log2 fold change ± 0.585, FDR <0.05) in one or more conditions versus sedentary, clustered based on Euclidean distance. Multimedia component 5

Multimedia component 6 Supplemental Figure 6. **Motif activity analysis clustering of genes affected in chromatin accessibility.** (**A**) Heatmap of motif activity (A.U) from ATAC-seq data, clustering based on Euclidean distances revealed annotated cluster 1. Multimedia component 6
