# Supplementary figures and images for: Endurance training promotes chromatin closure and timely repression of the post-exercise immediate early stress response
Source: Mol Metab. 2025 Jul 5;99:102206. doi: 10.1016/j.molmet.2025.102206 (PMC12309500; doi:10.1016/j.molmet.2025.102206)

**S1**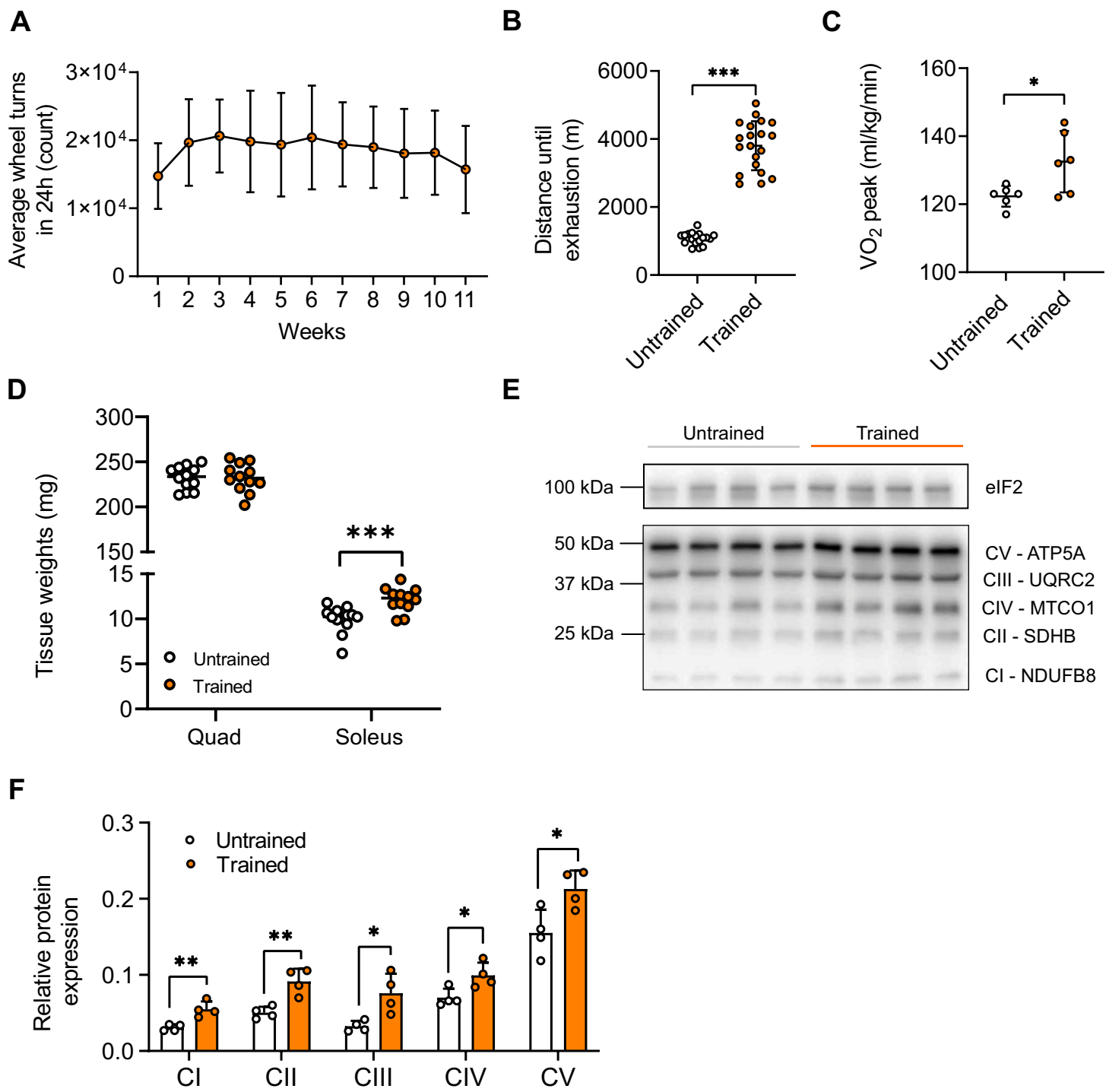

Supplement: Multimedia component 1 — Supplementary Figure 1. Adaptations induced by voluntary running wheel endurance training. (A) Number of voluntary running wheel turns in 24 h in trained animals. (B) Distance covered in maximal capacity test in meters. (C) VO2peak reached in a maximal capacity test. (D) Absolute mass for quadriceps (Quad) and soleus muscles of trained and untrained mice, averaged across both limbs. (E) Protein expression of electron transport chain subunits in quadriceps muscle. (F) Quantification of electron transport chain subunits expression, relative to loading control eIF2. In 1B-D: unpaired two-tailed t-test ∗p < 0.05, ∗∗∗<0.001 describe the training effect. [file mmc1.pdf]

A

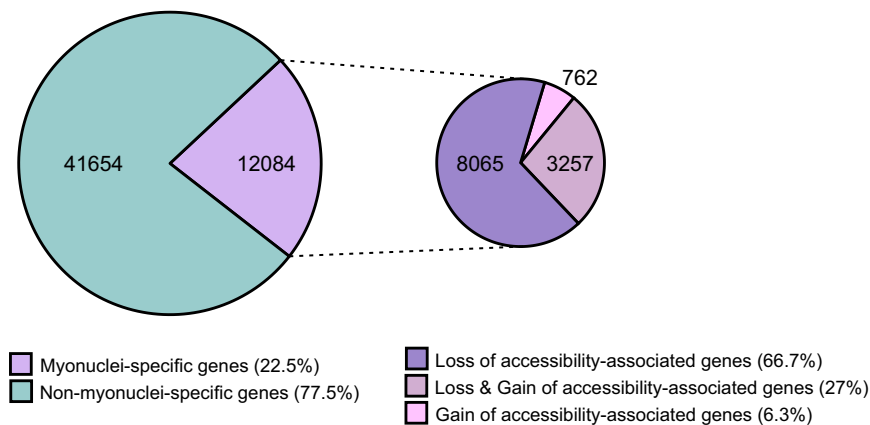

B

GO: Subset of 1848 genes exercise-induced up- or downregulation

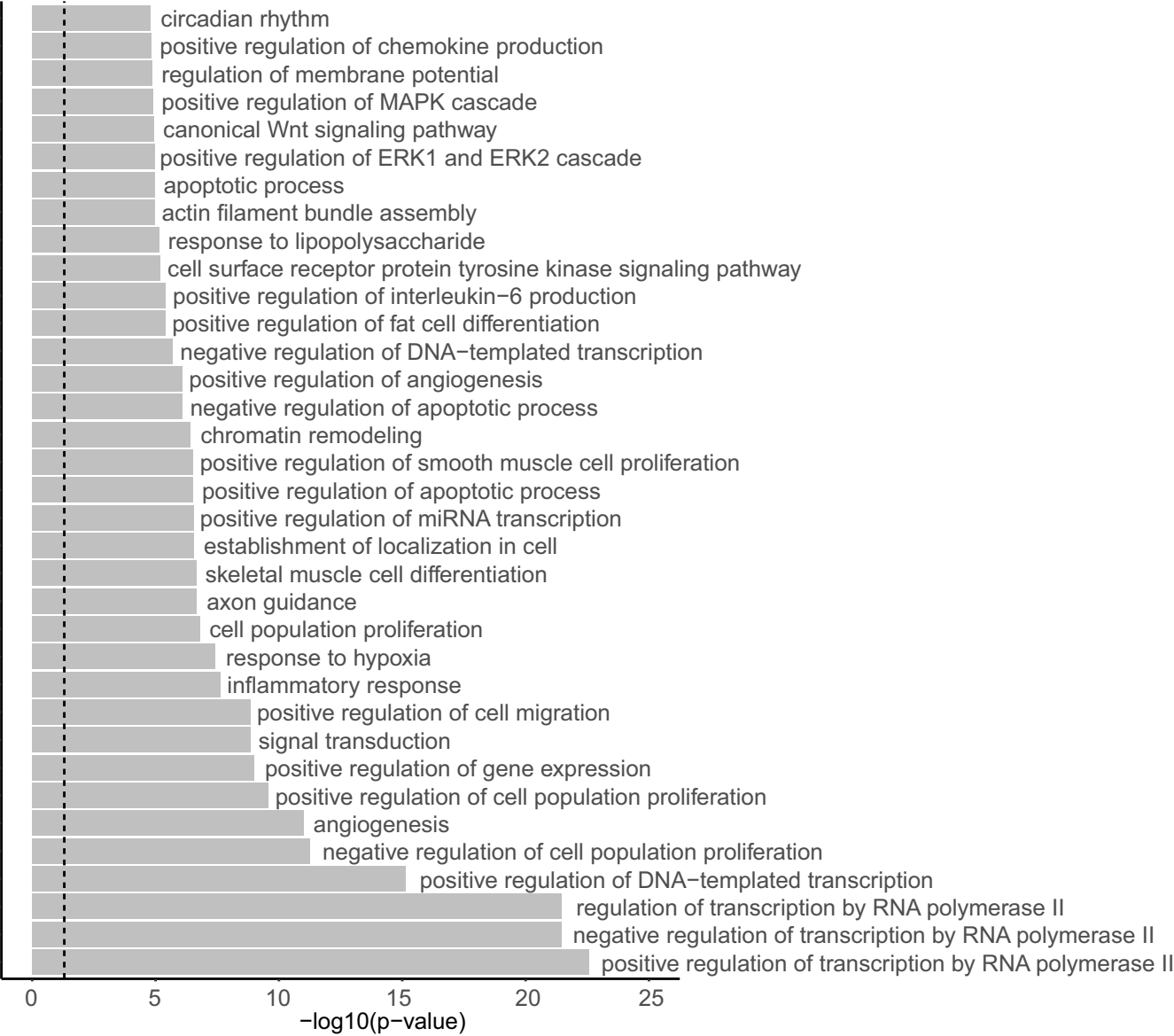

Supplement: Multimedia component 4 — Supplemental Figure 4. Proportions and functional annotation of transcripts associated to myonuclei-specific, exercise-induced genes from ATAC-seq data. (A) Pie chart representing the proportions of myonuclei (non-)specific genes from bulk RNA-seq count matrix, and the occurrence of these genes in gain and loss of accessibility list from ATAC-seq. Differential accessibility (loss & gain): every condition vs. sedentary, FDR<0.05. [file mmc4.pdf]

S5

A

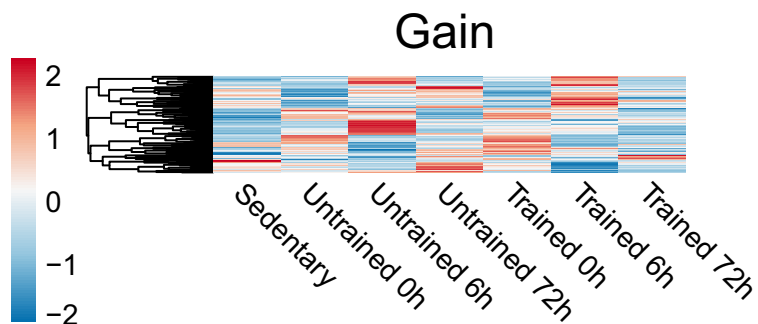

B

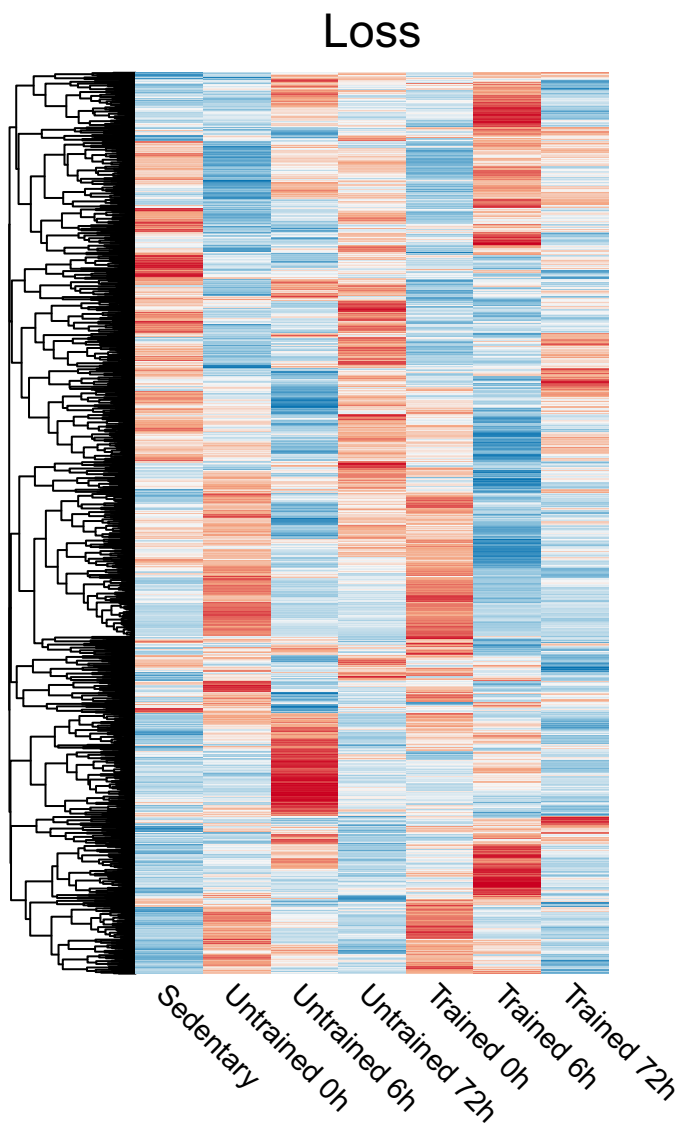

C

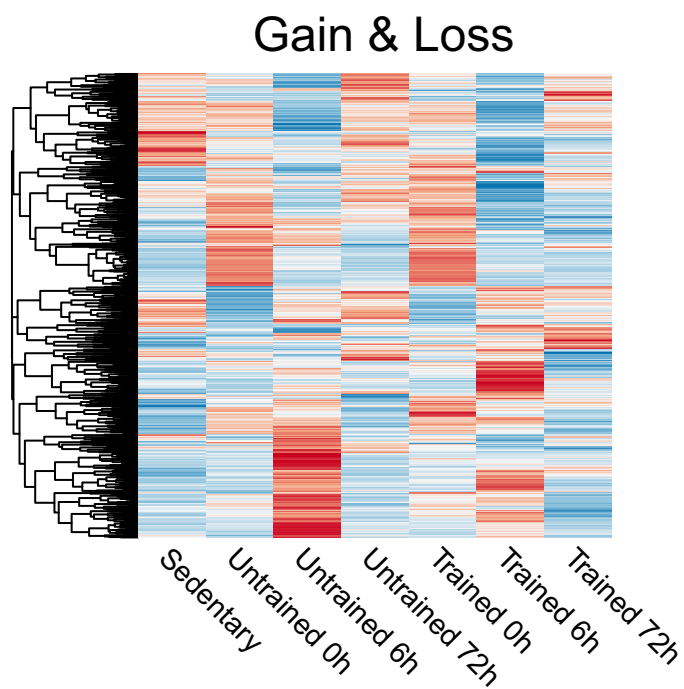

Supplement: Multimedia component 5 — Supplementary Figure 5. Correlation between chromatin opening/closing and gene expression changes. (A) Heatmap of z-scores [calculated as mean (log2 (TPM))] from gene expression data of genes associated with gain of chromatin accessibility in ATAC-seq data, that show significant up- or downregulation (log2 fold change ± 0.585, FDR <0.05) in one or more conditions versus sedentary, clustered based on Euclidean distance. (B) Heatmap of z-scores [calculated as mean (log2 (TPM))] from gene expression data of genes associated with loss of chromatin accessibility in ATAC-seq data, that show significant up- or downregulation (log2 fold change ± 0.585, FDR <0.05) in one or more conditions versus sedentary, clustered based on Euclidean distance. (C) Heatmap of z-scores [calculated as mean (log2 (TPM))] from gene expression data of genes associated with gain and loss of chromatin accessibility in ATAC-seq data, that show significant up- or downregulation (log2 fold change ± 0.585, FDR <0.05) in one or more conditions versus sedentary, clustered based on Euclidean distance. [file mmc5.pdf]

A

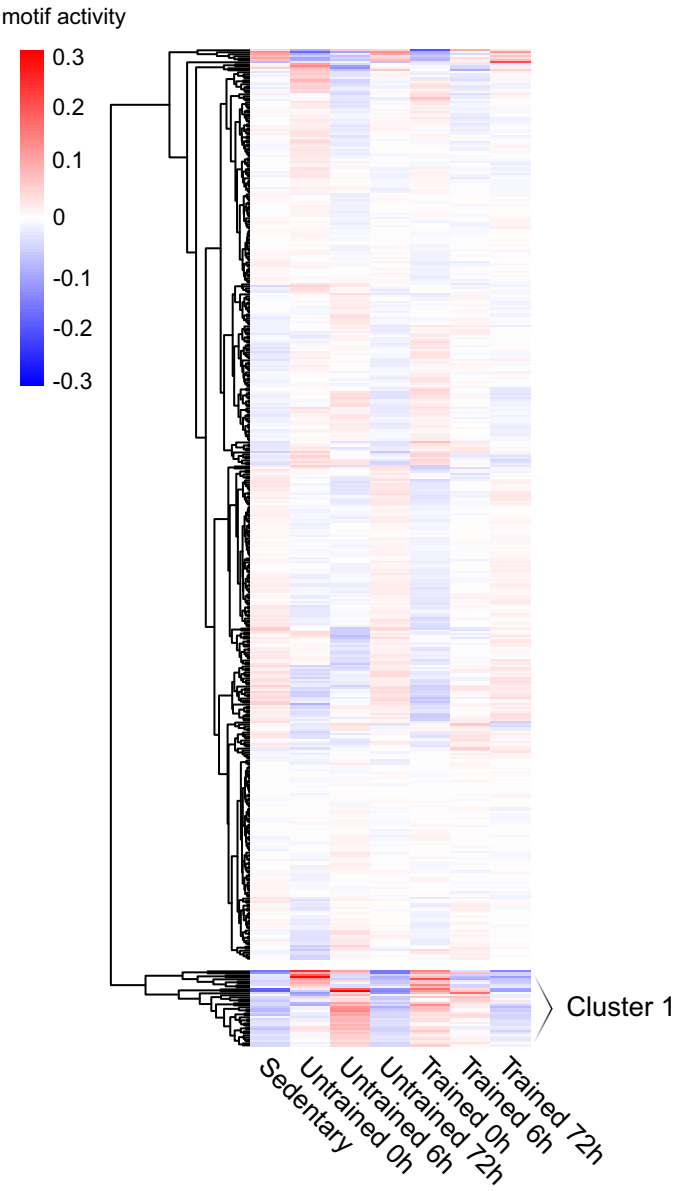

Supplement: Multimedia component 6 — Supplemental Figure 6. Motif activity analysis clustering of genes affected in chromatin accessibility. (A) Heatmap of motif activity (A.U) from ATAC-seq data, clustering based on Euclidean distances revealed annotated cluster 1. [file mmc6.pdf]
