# Supplementary material for: Endurance training promotes chromatin closure and timely repression of the post-exercise immediate early stress response
Source: Mol Metab. 2025 Jul 5;99:102206. doi: 10.1016/j.molmet.2025.102206 (PMC12309500; doi:10.1016/j.molmet.2025.102206)
Supplement: Multimedia component 2 — Supplemental Figure 2. Localization and differences in chromatin accessibility across conditions. (A) Distribution of differential chromatin accessibility across the genomic regions. The results depicted from sedentary animals is representative of similar data in the other experimental cohorts. (B) Pie chart representing the average distribution of genomic regions of differential chromatin accessibility across all conditions. (C) Venn diagram of genes associated to lost accessibility in untrained 0h and 6h. (D) Venn diagram of genes associated to gained accessibility in untrained 0h and 6h. (E) Venn diagram of genes associated to lost accessibility in trained 0h, 6h and 72h. (F) Venn diagram of genes associated to gained accessibility in trained 0h, 6h and 72h. [file mmc2.pdf]

S2

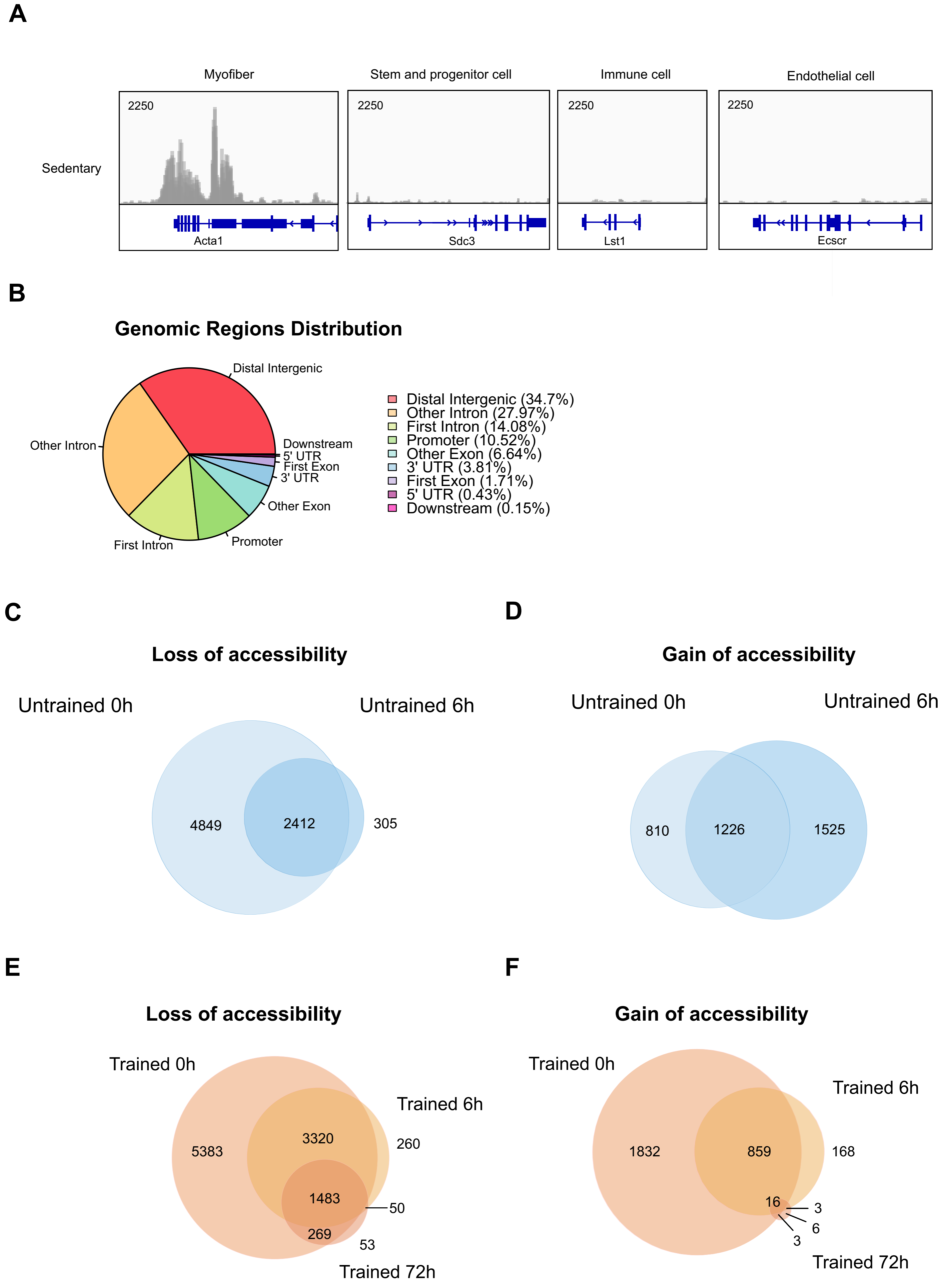
A
Myofiber
Stem and progenitor cell
Immune cell
Endothelial cell
Sedentary
2250
2250
2250
2250
Acta1
Sdc3
Lst1
Ecscr
B
Genomic Regions Distribution
Distal Intergenic
Other Intron
Downstream
5' UTR
First Exon
3' UTR
Other Exon
Promoter
First Intron
Distal Intergenic (34.7%)
Other Intron (27.97%)
First Intron (14.08%)
Promoter (10.52%)
Other Exon (6.64%)
3' UTR (3.81%)
First Exon (1.71%)
5' UTR (0.43%)
Downstream (0.15%)
C
Loss of accessibility
Untrained 0h
Untrained 6h
4849
2412
305
D
Gain of accessibility
Untrained 0h
Untrained 6h
810
1226
1525
E
Loss of accessibility
Trained 0h
Trained 6h
5383
3320
260
1483
50
269
53
Trained 72h
F
Gain of accessibility
Trained 0h
Trained 6h
1832
859
168
16
3
6
3
Trained 72h
