# Supplementary material for: Endurance training promotes chromatin closure and timely repression of the post-exercise immediate early stress response
Source: Mol Metab. 2025 Jul 5;99:102206. doi: 10.1016/j.molmet.2025.102206 (PMC12309500; doi:10.1016/j.molmet.2025.102206)
Supplement: Multimedia component 3 — Supplemental Figure 3. Characterization of chromatin accessibility changes (A) Top 15 Gene Ontology (GO) biological process (BP) terms, enriched in genes chromatin accessibility loss at 0h, only in untrained. (B) Venn diagram of genes associated to lost accessibility in untrained 0h, and gained accessibility in trained 0h. (C) Top 15 GO BP terms enriched in genes of the overlap between untrained 0h-lost and trained 0h-gain of chromatin accessibility. (D) Venn diagram of genes associated to gained accessibility in untrained 6h, and lost accessibility in trained 6h. (E) Top 15 GO BP terms enriched in genes of the overlap between untrained 6h-gained and trained 6h-loss of chromatin accessibility. In S2C: Differential accessibility (loss & gain): every condition vs. sedentary, FDR<0.05. In S2B, D: Dashed line indicates a significance threshold of P = 0.05. [file mmc3.pdf]

S3

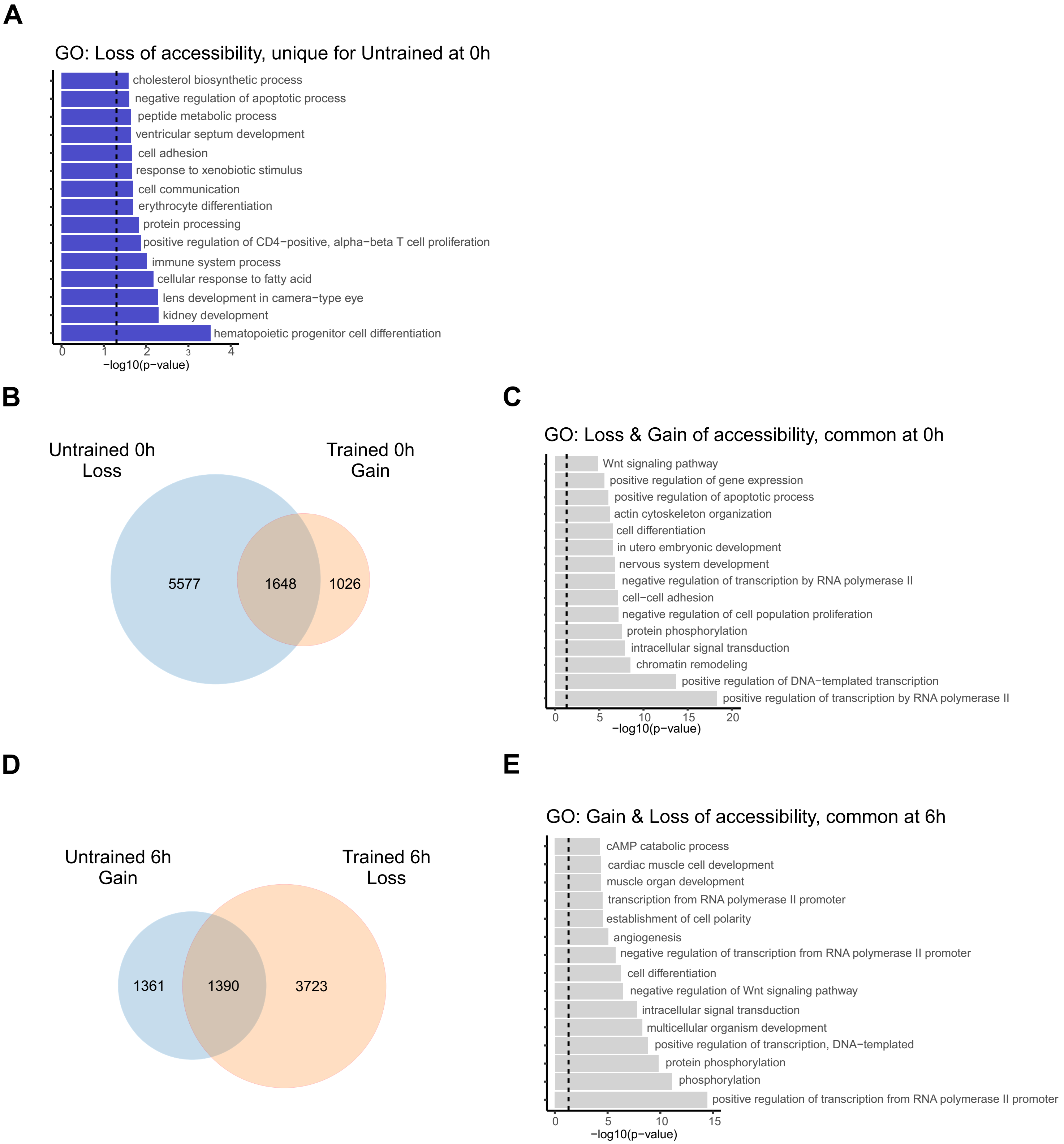
A
GO: Loss of accessibility, unique for Untrained at 0h
cholesterol biosynthetic process
negative regulation of apoptotic process
peptide metabolic process
ventricular septum development
cell adhesion
response to xenobiotic stimulus
cell communication
erythrocyte differentiation
protein processing
positive regulation of CD4−positive, alpha−beta T cell proliferation
immune system process
cellular response to fatty acid
lens development in camera−type eye
kidney development
hematopoietic progenitor cell differentiation
0
1
2
3
4
−log10(p−value)
B
Untrained 0h
Loss
Trained 0h
Gain
5577
1648
1026
C
GO: Loss & Gain of accessibility, common at 0h
Wnt signaling pathway
positive regulation of gene expression
positive regulation of apoptotic process
actin cytoskeleton organization
cell differentiation
in utero embryonic development
nervous system development
negative regulation of transcription by RNA polymerase II
cell−cell adhesion
negative regulation of cell population proliferation
protein phosphorylation
intracellular signal transduction
chromatin remodeling
positive regulation of DNA−templated transcription
positive regulation of transcription by RNA polymerase II
0
5
10
15
20
−log10(p−value)
D
Untrained 6h
Gain
Trained 6h
Loss
1361
1390
3723
E
GO: Gain & Loss of accessibility, common at 6h
cAMP catabolic process
cardiac muscle cell development
muscle organ development
transcription from RNA polymerase II promoter
establishment of cell polarity
angiogenesis
negative regulation of transcription from RNA polymerase II promoter
cell differentiation
negative regulation of Wnt signaling pathway
intracellular signal transduction
multicellular organism development
positive regulation of transcription, DNA−templated
protein phosphorylation
phosphorylation
positive regulation of transcription from RNA polymerase II promoter
0
5
10
15
−log10(p−value)
